# Differentiation of primordial germ cells from premature ovarian insufficiency-derived induced pluripotent stem cells

**DOI:** 10.1186/s13287-019-1261-6

**Published:** 2019-05-31

**Authors:** Sheng Yang, Shufang Ding, Shiwei He, Lixia He, Kefei Gao, Shuping Peng, Cijun Shuai

**Affiliations:** 1 The Center of Reproduction Medicine, The University of Hong Kong-Shenzhen Hospital, Shenzhen, 518053 Guangdong Province People’s Republic of China; 2 Department of Obstetrics and Gynecology, Shenzhen University General Hospital, Shenzhen University Clinical Medical Academy, 518055, Shenzhen, Guangdong Province People’s Republic of China; 3 The Key Laboratory of Carcinogenesis and Cancer Invasion of the Chinese Ministry of Education, the Key Laboratory of Carcinogenesis of The Chinese Ministry of Health and Cancer Research Institute, Xiangya Hospital, Central South University, Changsha, 410078 People’s Republic of China; 4 State Key Laboratory of High Performance Complex Manufacturing, Central South University, Changsha, 410083 People’s Republic of China; 5 Jiangxi University of Science and Technology, Nanchang, 330013 People’s Republic of China

**Keywords:** Premature ovarian insufficient, Induced pluripotent stem cells, Primordial germ cells, Reprogramming, Differentiation

## Abstract

**Background:**

Premature ovarian insufficiency (POI) is a common disease in reproductive women. The pathogenesis of POI is not clear, although it is known that it involves the disorder of oocyte differentiation and development. The introduction of reprogramming human somatic cells into induced pluripotent stem cells (iPSCs) offers a unique opportunity to study many aspects of POI from cell differentiation in vitro that could ultimately lead to novel drug development and testing to help treat the disorder.

**Methods:**

The fibroblasts from POI patients, including fragile X syndrome, abnormal karyotype (45, X; 45, X/46, XX; 45, XO and 47, XXX), and the gene mutation (*FIGLA* and *GDF9*) were reprogrammed to pluripotency status by retroviral transduction using defined factors. The morphology, growth characteristics, gene expression profiles, epigenetic status, and in vitro and in vivo differentiation potential of the POI-1-iPSCs (from fragile X syndrome) were analyzed. Then, POI-1-iPSCs were induced to differentiation into primordial germ cells (PGCs) with DNA methyltransferase inhibitors.

**Results:**

The iPSCs were successfully generated from POI patients’ fibroblasts. The formed iPS clones have the same characteristics of human ESCs. POI-1-iPSCs were successfully generated with germline competence. The POI-1-iPSCs, with genotypes of fragile X syndrome, can be induced to differentiation into PGCs with high efficiency under our culture system by DNA demethylation. This study proved that disease-specific iPSC lines derived from POI patients could be generated and successfully differentiated into PGCs.

**Conclusions:**

We established some novel, systemic cell models for the studying of the pathogenesis of POI patients. Second, DNA demethylation may accelerate the induction of human PGCs from iPSCs in vitro and the conclusion needs further exploration. This represents an important step in the novel approach for the study of the pathophysiology and potential egg resource for POI patients.

**Electronic supplementary material:**

The online version of this article (10.1186/s13287-019-1261-6) contains supplementary material, which is available to authorized users.

## Introduction

Premature ovarian insufficiency (POI) is a complex disorder that seriously affects the fertility of women of reproductive age. In reproductive women, the incidence of POI is approximately 1–3% [1]. POI leads to infertility and metabolic abnormality. The detailed mechanisms behind POI are mostly unknown. The development of POI is due to follicle dysfunction or follicle depletion [2]. The causes of the POI are damage to the ovary, autoimmune disease, and family genetics, which includes X chromosome abnormality, a major cause of POI [3, 4]. Exploring the cause of POI requires an understanding of the oocyte maturation process and how the inherited genetic defect affects this process. However, very few POI studies have focused on the oocyte maturation process. Due to restrictions of the researches about human embryos and less access to human embryos with inherited POI, we performed the pathological study of POI difficultly with a visual angle of dynamics and continuous cell and embryo development. Thus, an effective cell model is essential for exploring the pathogenesis of POI at different stages of follicle development.

The human male germ cell can be successfully induced from iPS/ES cells in vitro and fully differentiated into spermatocytes in vivo [5–7]. Unlike male germ cells, the differentiation of human oocytes from stem cells has not been fully addressed. Although oocytes from iPSCs/ESCs have been established in mice [8], the details of the oocyte maturation process in vitro are still not well defined. Furthermore, unlike in mice, human PGCs derived from human iPSCs/ESCs cannot be directly induced. It was believed that human ESCs are similar to epiblast-like cells (EpiLCs) in mice [9, 10] and do not have a fully pluripotent status [11]. A recent study used a new induction system (4i culture system) to induce human iPSCs/ESCs to show the distinct epigenetic landscape, characterized by the depression of preimplantation epiblast genes [12]. The use of human iPSCs generated by such methods in 4i culture system is likely to facilitate the subsequent appropriate differentiation pathway to germ cells, as demonstrated by two mouse experiments in which mouse pluripotent stem cells were differentiated into germ cells [8]. In addition, a recent study showed that Sox17 regulated the fate of human PGCs; nevertheless, BLIMP1 repressed the expression of endodermal and other somatic genes during the differentiation of human PGCs. The molecular mechanism differences between mouse and human PGC specification may lead to their divergent embryonic development and pluripotent states, which might affect other early cell-fate decisions [13]. This result also offered us insight into understanding the basis of the progression of pluripotent stem cells to PGCs. We hope to further improve the methods to establish human PGCs from human POI-iPSCs.

Here, we apply and improve on previous methods to establish human POI-iPSC lines using 4 inhibitor culture systems from 7 POI patients with a family history, including Fragile X syndrome, abnormal karyotype (45, X; 45, X/46, XX; 45, XO and 47, XXX), and the gene mutation (*FIGLA and GDF9*). We successfully explored their differentiation potential for germ cells and mimicked the in vitro differentiation of human PGCs in POI patients.

## Materials and method

### The diagnostic criteria and the characteristics of the POI patients

In our study, the POI patients with a family history were diagnosed by primary or secondary amenorrhea for a 4- to 6-month duration before the age of 40 years. Additionally, for these individuals, anti-Mullerian hormone (AMH) levels are low (0.5 ng/ml), while LH and FSH levels are elevated (FSH: 40 mIU/ml). According the criteria, 7 patients with POI, including Fragile X syndrome, abnormal karyotype (45, X; 45, X/46, XX; 45, XO and 47, XXX), and the gene mutation (*FIGLA* and *GDF 9*) were selected. The clinical features, including age, hormone levels, and genetic characteristics of patients in the POI and the no-POI groups were summarized in Additional file 1: Table S1.

### The establishment of POI-derived iPSCs

The adult cells were collected and reprogrammed from the POI patients with a familial inheritance as described previously [13, 14]. In short, the iPSCs were grown on irradiated mouse embryonic fibroblasts (MEFs) in N_2_B_27_ medium (Invitrogen) supplemented with 100 U/ml LIF (ESGRO Millipore), 3 mM GSK3 inhibitor CHIR99021 (Axon Medchem BV), SB203580 (TOCRIS bioscience), 5 mM SP600125 (TOCRIS bioscience), and 1 mM MEK inhibitor PD0325901 (Stemgent). POI-derived iPSCs were trypsinized using 0.05% trypsin-EDTA (Sigma) into single cells and plated into new wells with a mouse EF feeder every 3 or 4 days.

### Immunocytochemistry and alkaline phosphatase staining

Undifferentiated POI-derived iPSC clones were fixed for 30 min in 4% paraformaldehyde at room temperature and then permeabilized with Triton X-100 for 30 min. After 30 min of blocking with 5% fetal bovine serum in PBS with 0.1% Tween-20, the cells were stained with primary antibodies overnight at 4 °C. Then, the stained cells were incubated with secondary antibodies (1 h at a dilution of 1:200). After staining, the cells were analyzed using confocal laser-scanning microscopy (Zeiss). The alkaline phosphatase activity was detected using alkaline phosphatase detection kit (Millipore, Billerica, USA). Briefly, at room temperature, human iPS cells were fixed with 4% paraformaldehyde for 2 min. The cells were washed three times with TBST buffer and stained with a naphthol/Fast Red Violet solution (a mixture of Fast Red Violet, naphthol AS-BI phosphate solution, and water in a 2:1:1 ratio) for 15 min in the dark. The cells were rinsed with TBST and covered with PBS. Alkaline phosphatase-positive cells were imaged using a light microscope.

### In vitro and in vivo differentiation assay

For in vitro and in vivo differentiation, the details were as described previously [14]. Briefly, embryonic bodies were formed then transferred onto a plate for adherent culture for 15 days in EB medium, followed by immunocytochemistry analysis. For in vivo differentiation assay, the POI-iPSCs were mechanically cut into small clumps (approximately 100 cells each). Approximately 0.5–1 × 10^6^ cells were collected and injected into the rear leg of 6- to 8-week-old NOD/SCID mice. Ten weeks later, the tumors were harvested and processed for hematoxylin and eosin (H&E) staining.

### Real-time PCR analysis

The total RNA was extracted from 1 × 10^6^ iPSCs. Then, 4-μl aliquots of the total RNA were reverse-transcribed using the Revert-Aid™ First Strand cDNA Synthesis Kit (Fermentas, Lithuania, USA). The primers used for real-time RT-PCR are shown in Additional file 1: Table S1. Real-time RT-PCR was performed using a Light Cycler Fast Start Master DNA SYBR Green I Kit (Roche, Germany). Afterward, a melting curve was generated with no nonspecific products. To determine the ∆Ct value, the cycle threshold (Ct) values were obtained and the relative gene expression level was calculated. Using the 2^-∆∆Ct^ method and a 95% accepted confidence interval, the relative change was calculated.

### Differentiation and induction of primordial germ cells from POI-derived iPSCs

The detailed protocol for stem cell culture and PGC differentiation is mainly based on paper described by Yang et al. [13]. The main procedure is included pre-induction and PGC induction. In our culture system, the DNA methyltransferase inhibitor (DNMTi) 5-aza-2′-deoxycytidine (DAC) (Sigma-Aldrich, Taufkirchen, Germany) is added after pre-induction for demethylation at day 5. The final centration was 0.05 uM. These cells were cultured 2 days with DAC and PGC medium for downstream experiments.

Stock solutions (1 mM) of DAC was dissolved in DMSO. Aliquots were stored at − 80 °C and diluted prior to each treatment in fresh PGC induction medium to the required concentrations, keeping the final concentration of DMSO (0.005%) non-toxic to the cells.

### Bisulfite sequencing

Genomic DNA was extracted using a genomic DNA purification kit (QIAGEN). Two hundred nanograms of genomic DNA from each sample was treated with a Methylamp DNA modification kit (P-1001-1, Epigentek, Brooklyn, NY, USA) to convert the unmethylated cytosine to uracil according to the manufacturer’s instructions. The three imprinted genes *H19*, *HEG1*, and *SNRPN* amplified by PCR using EX Taq HS (Biomed), cloned into the PCR 2.1 vector (Invitrogen), and sequenced with M13. PCR primers were summarized in Additional file 2: Table S2.

### Statistical analyses

Statistical analyses were performed using SPSS version 21.0. The data are presented as the means ± SEM and were analyzed by *t* tests. A *P* value of < 0.05 was considered statistically significant.

## Results

### The establishment of iPSCs from the adult cells of POI patients

**Fig. 1:**
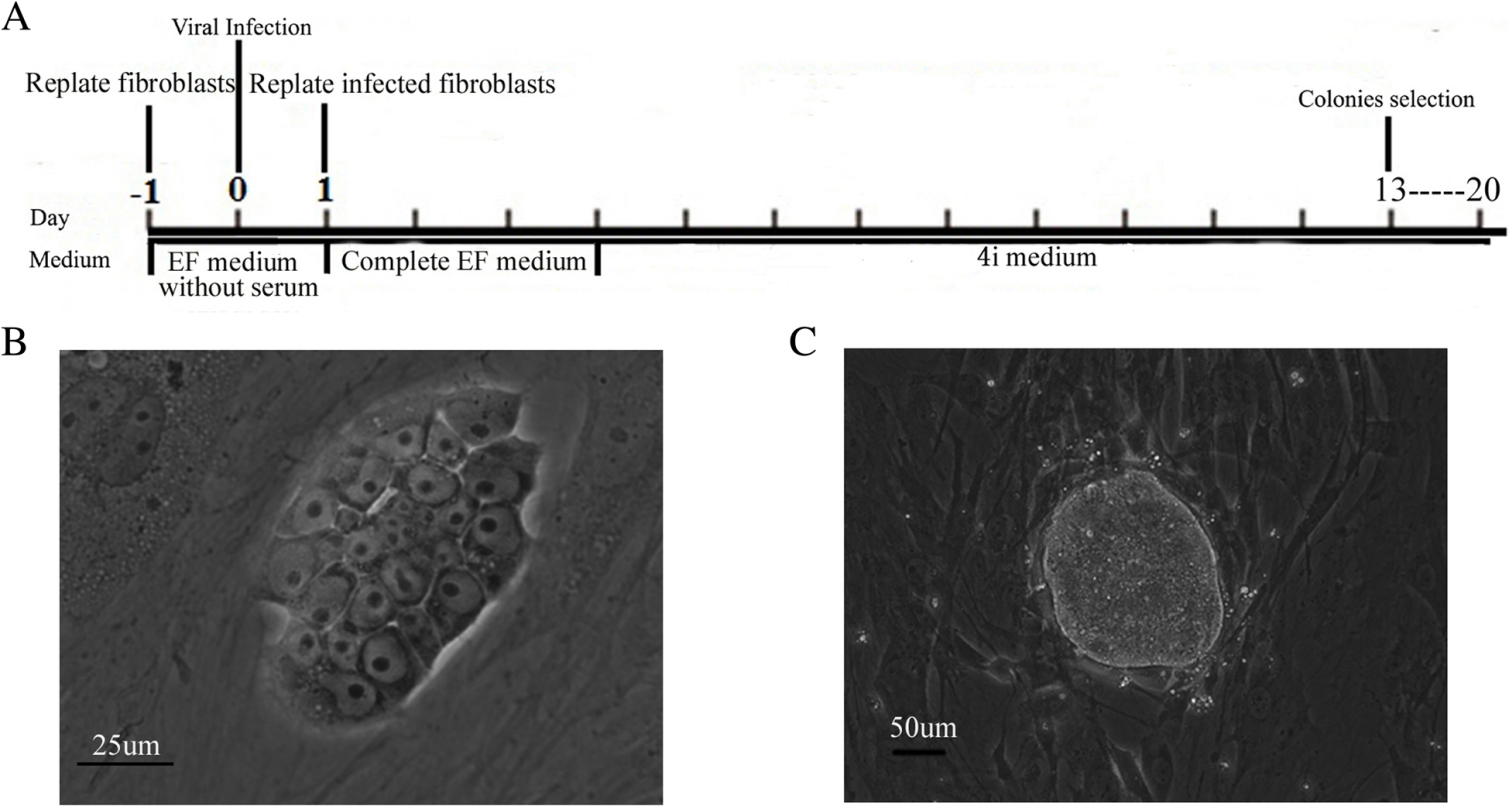
The generation of POI-iPSCs from adult fibroblasts. **a** The timeline of virus-mediated reprogramming in adult cells of POI patients. **b** The morphology of POI-iPS clones at an early stage. **c** The typical POI-iPS clones at day 25

Adult epithelial cells in urine were used for reprogramming under informed consent. The time schedule for POI iPS induction is summarized (Fig. 1a). After two passage culture in serum-free medium, the cells were transduced with lentiviral vectors with four factors, including *Oct4*, *Sox2*, *Klf4*, and *c-myc* at day 0. At day 1, the transfected cells were removed from the feeder cells with complete EF culture medium. At day 4, the medium was changed to 4i medium for further induction. At approximately days 14 to 20, ES cell-like colonies formed with high nucleus-to-cytoplasm ratios and prominent nucleoli (Fig. 1b). The POI-iPSCs became compact with continuous culture (Fig. 1c). Then, these colonies were cultured and expanded on the feeder cells mouse embryonic fibroblasts.

In our study, 7 POI patients were diagnosed and were selected for reprogramming. Ten iPS cell lines were established from these patients by these methods, and 6 POI-derived iPS cell lines were from three same patients. Meanwhile, 2 iPS cell lines were also established from no POI patients as a control. All POI iPSC cell lines and no POI iPSCs were identified and proved that these iPSC cell lines were pluripotency. The POI-1-iPSC lines with Fragile X syndrome were then subsequently propagated and characterized in more details. More details about cell lines see Additional file 1: Table S1. At the same time, we detected the karyotype and targeted sequencing in iPSCs and PGCLCs during the reprogramming and differentiation. More details about the disease-associated genotype please see Additional file 3.

### The generated POI-1-iPSCs showed similar characteristics as human ESCs

**Fig. 2:**
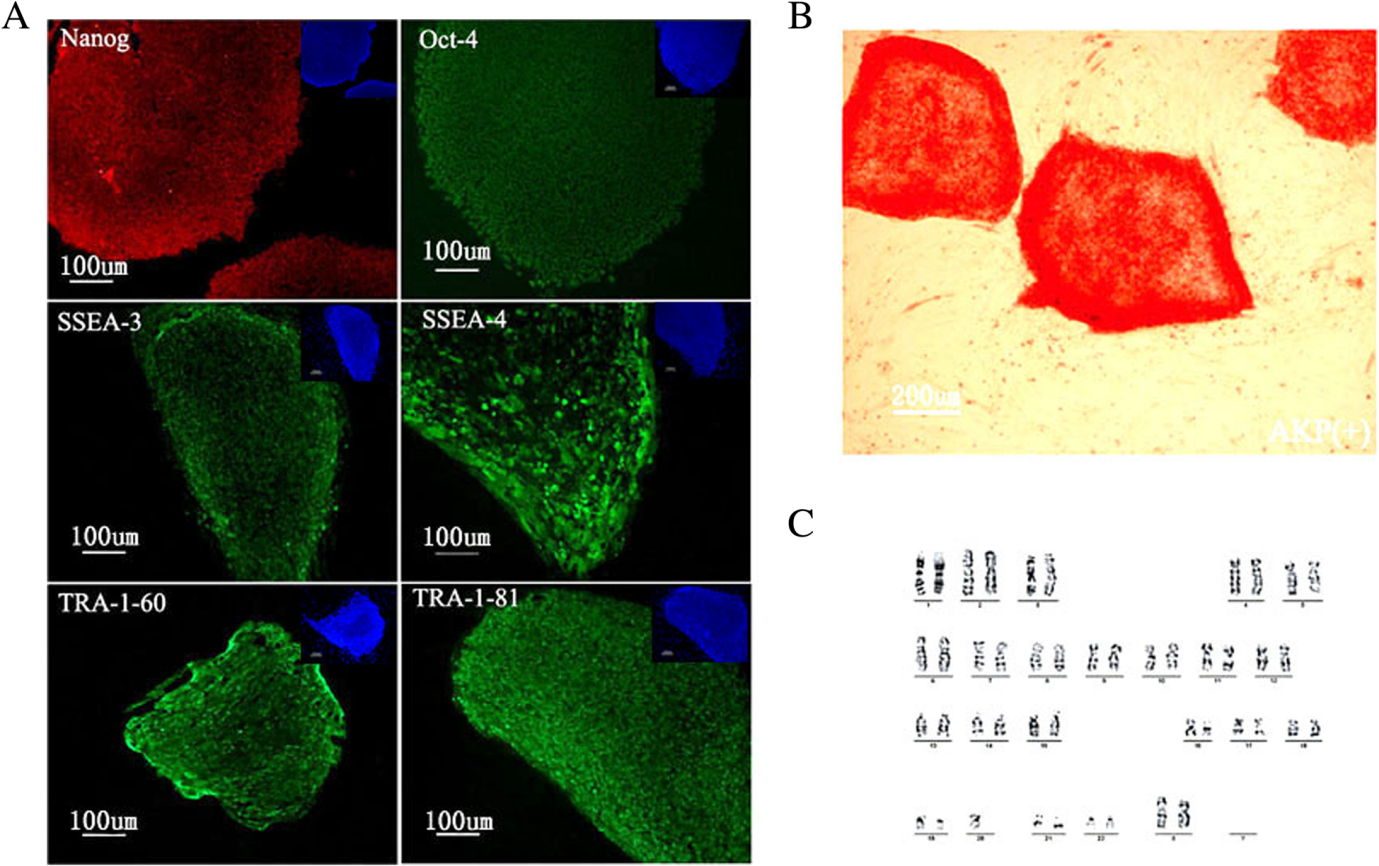
POI-1-iPSCs showed the characteristics of human ESCs. **a** All colonies stained positive for OCT4, NANOG, SSEA-3, SSEA-4, TRA-1-60, and TRA-1-81. **b** POI-1-iPSCs were AKP positive. **c** The G-banding of POI-1-iPSCs at passage 20 showed a normal karyotype

Human POI-1-iPSC clones exhibited growth morphology similar to that of human ESCs. POI-1-iPS cell clones were positive for OCT4, NANOG, SSEA-3, SSEA-4, TRA-1-60, and TRA-1-81 and negative for SSEA-1, as indicated by immunofluorescence (Fig. 2a), and also positive for alkaline phosphatase (Fig. 2b). After reprogramming, the karyotype of the POI-1-iPSCs was normal (Fig. 2c). The special gene markers, such as *Oct-4*, *Nanog*, *Sox-2*, *TERF1*, *TERF2*, *REX*, *FGF4*, *Cripto*, *Thy1*, and *LEFTYA* were expressed in POI-1-iPSCs (data not shown). In order to evaluate whether the vector transgenes was working in derived iPS cell clones, the transgene-specific transcripts of OCT4, KLF4, and c-MYC were performed using RT-PCR. The presence of exogenous pluripotency genes was evident in most of the clones (data not shown). The morphology of clones and expression of pluripotent genes indicated the establishment of POI disease-specific iPSCs.

### POI-1-iPSCs have the potential to differentiate into three germ layers

**Fig. 3:**
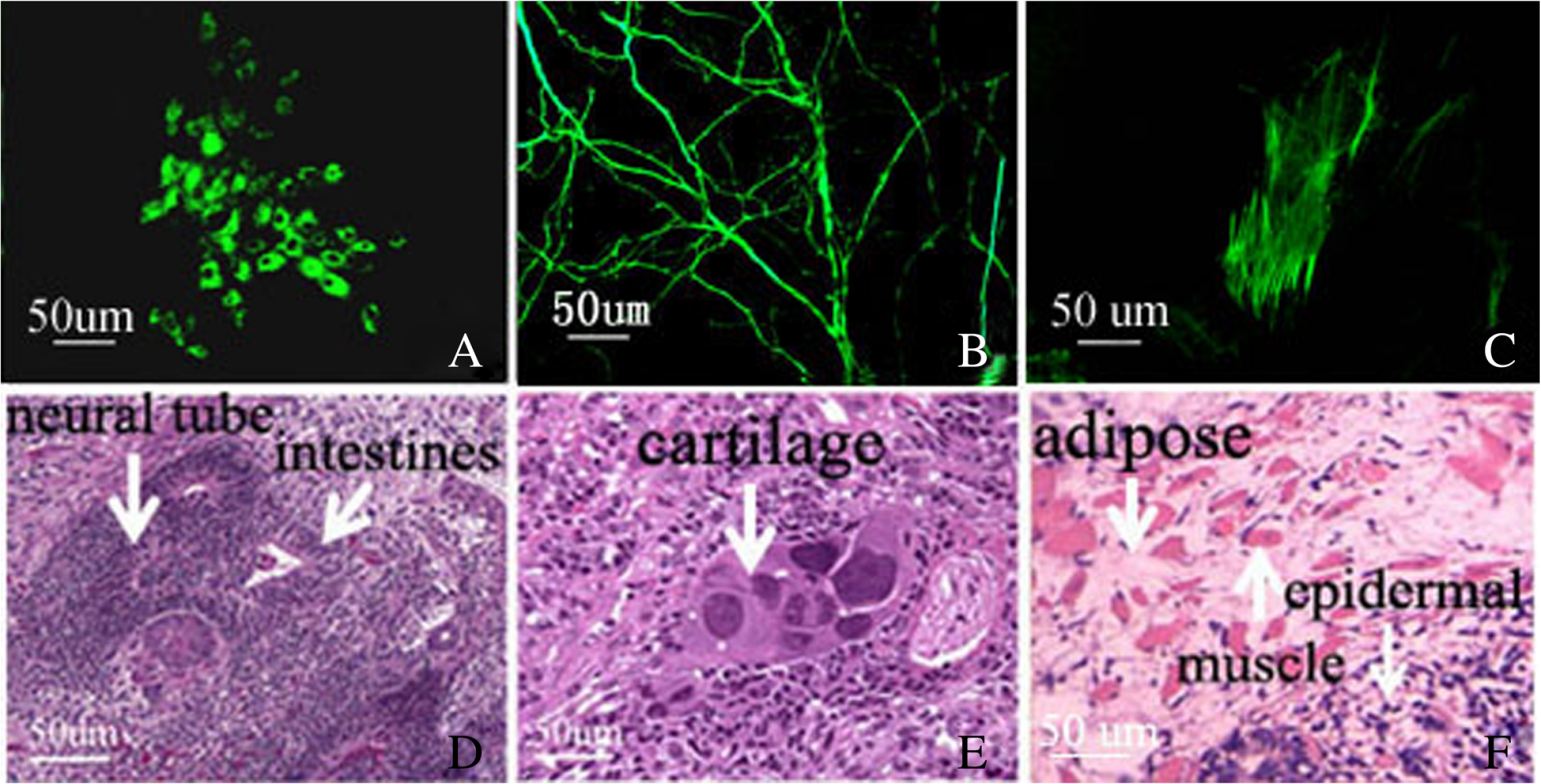
POI-1-iPSCs differentiate into all three germs in vitro and in vivo. **a** POI-1-iPSCs were induced to express AFP. **b** POI-1-iPSCs were induced to differentiate into smooth muscle. **c**. POI-1-iPSCs were induced to differentiate into nerve cells. Teratoma formation indicated the existence of tissues from all three germ layers, such as **d** intestines (endoderm) (ectoderm), neural tube (ectoderm), **e** cartilage (mesodermal), and **f** adipose tissue (mesoderm), epidermal tissues and muscle (mesoderm)

To evaluate their differentiation potential, we cultured POI-1-iPSCs and then formed well-shaped EBs. After 7 day culture in suspension status, EBs were removed to new dishes and continuously cultured in bFGF free ES medium. Various cell types were observed in the outgrowth over 15 days. Feta-protein (endoderm) (Fig. 3a), ß-tubulin (ectoderm) (Fig. 3b), and smooth muscle antigen (mesoderm) (Fig. 3c) were expressed in these cells by immunocytochemistry analysis showed the potential to differentiate into three germ layers. To assess the differentiation potential of POI-1-iPSCs in vivo, POI-1-iPSCs from 3 different iPS cell lines and different patients were injected into SCID-beige mice for teratoma formation. Four weeks after injection, 6 well-shaped teratomas were formed. Histochemical results showed the presence of some tissues, such as a neural tube, pigment retinal epithelium, and epidermal tissues (ectoderm) (Fig. 3d), cartilage, adipose tissue (mesoderm), muscle (mesoderm) (Fig. 3e), and intestines (endoderm) (Fig. 3f). At the same time, our results also showed that POI-1-iPSCs at either early (P8) or late (P25) passages could form teratomas indistinguishably containing derivatives of three germ layers. These results showed that the 10 established POI-1-iPSCs were pluripotent status and had the potential of differentiation into three germ layers.

### The differentiation of PGCs from POI-1-iPSCs

**Fig. 4:**
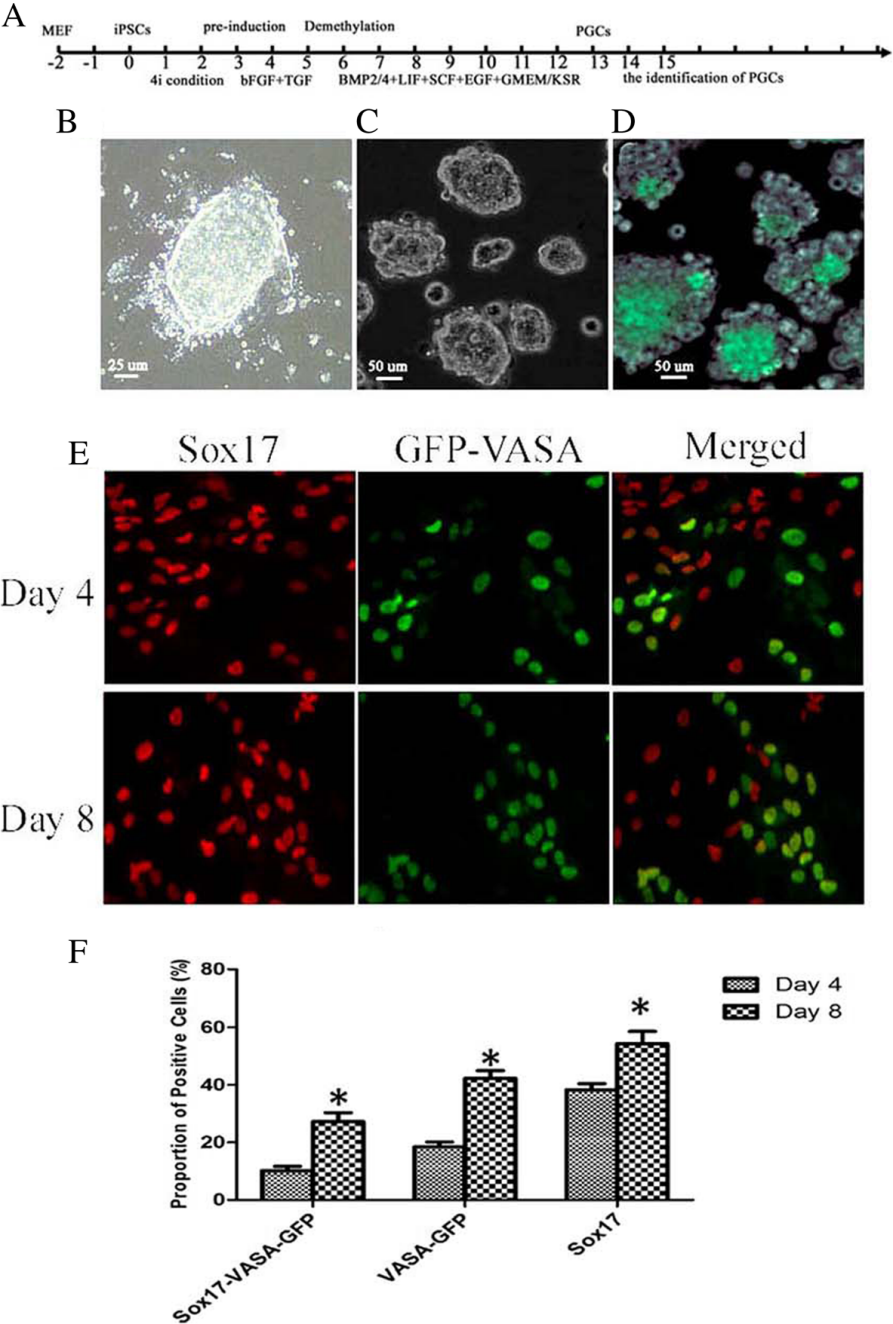
POI-1-iPSCs differentiate into primordial germ cells. **a** The timeline of PGC induction from POI-1-iPSCs. **b** The VASA-GFP POI-1-iPSCs were induced and kept in 4i culture medium. **c** For pre-induction, VASA-GFP POI-1-iPSCs were dissociated and cultured in pre-induction medium. **d** After day 8 of PGC induction, some cells expressed VASA-GFP. **d** At day 4 and 8 of PGC induction, Sox17 and VASA-GFP were both expressed in induced cells. With further induction, more cells expressed Sox17 and VASA. **e** The Sox17 and GFP-VASA double staining cells between day 4 and day 8 in the induced system, indicating statistical difference among them (Scale bars=50µm). **f** There is statistically difference in Sox17, VASA-GFP and Sox17-VASA-GFP double staining cells betwwen Day 4 and Day 8

As VASA is the most reliable marker for distinguishing human PGCs from iPSCs and ESCs, we first constructed the special PGC gene reporter system VASA-GFP. To monitor the differentiation of PGCs, the special PGC gene reporter VASA-GFP was transferred into POI-1-iPSCs, and iPSCs-11, which is from no POI patients. The stable POI-1-iPSCs and VASA-GFP iPSCs-11 were shown to be pluripotent by detecting the gene expression of the pluripotent marker, the differentiation potency, and the karyotype after transferring. As described in the methods section, the time schedule for PGC induction differentiation in our study is summarized (Fig. 4a). The differentiation of PGCs was performed in two stages, including pre-induction and PGC induction. DNA methyltransferase inhibitor (DNMTi) was added at day 5 after pre-induction. To maintain the full pluripotent status, the VASA-GFP POI-1-iPSCs were induced and kept in 4i culture medium (Fig. 4b). For pre-induction, VASA-GFP POI-1-iPSCs were dissociated and cultured in pre-induction medium (Fig. 4c). After 4 days PGC induction, some cells were VASA-GFP positive (Fig. 4d). Sox17 is the key regulator of human PGC-like fate. To detect the specification of human PGCs from POI-1-iPSCs, the level of Sox17 and VASA-GFP was analyzed by immunohistochemistry. At days 4 and 8 of PGC induction, Sox 17 and VASA-GFP were both expressed in induced cells. With further induction, more cells expressed sox17 and VASA (Fig. 4e). Very importantly, more cells expressed both, indicating the specification of human PGCs from POI-1-iPSCs. There is a statistical difference in Sox17 and VASA-GFP double staining cells between day 4 and day 8 in our experiments (Fig. 4f). As a control group, these experiments were performed using the normal iPSCs-11. Our results also showed the same differentiation characteristics as POI-1-iPSCs during PGC induction (data not shown).

**Fig. 5:**
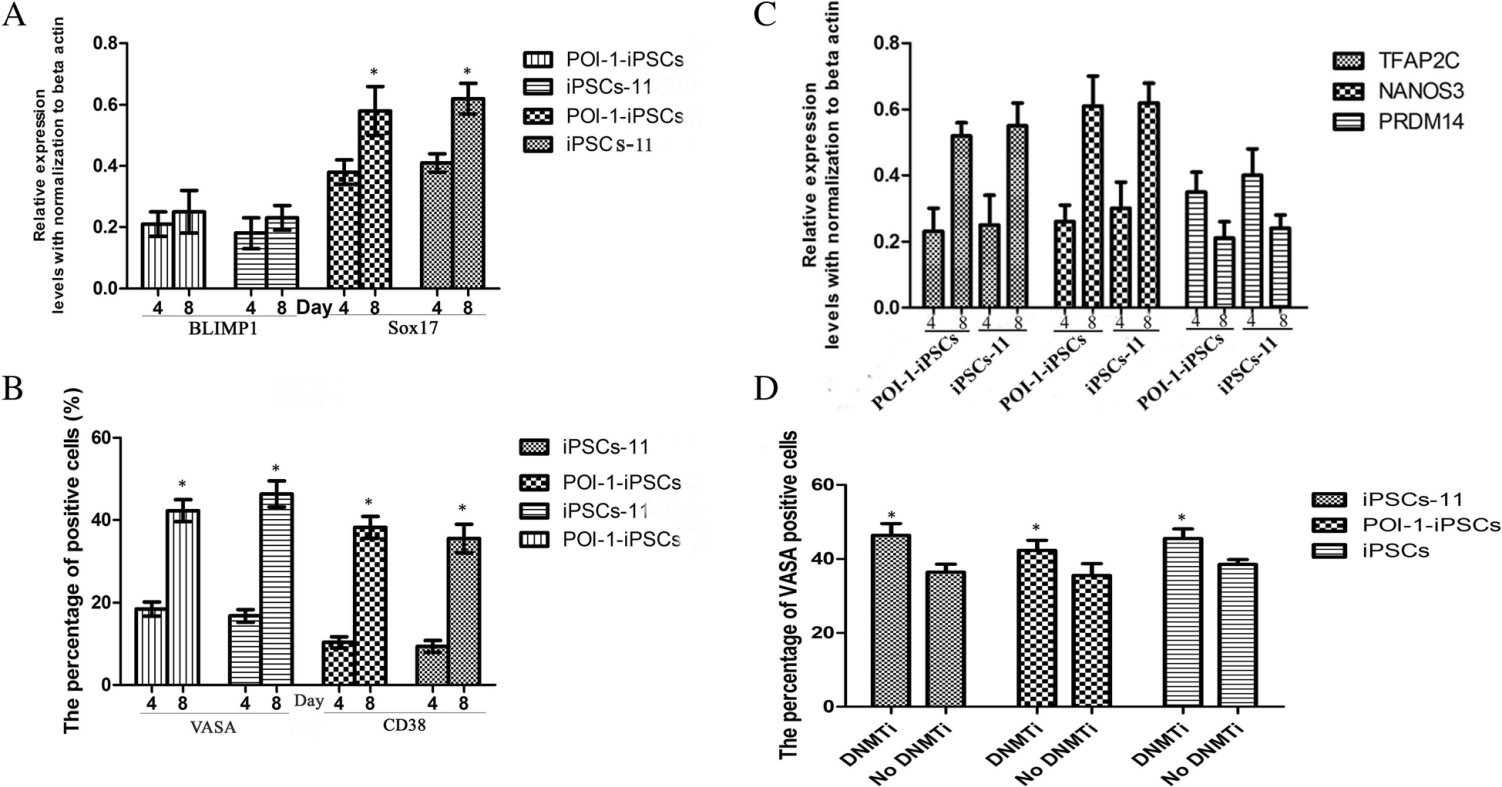
PGCs from POI-1-iPSCs during the late stage of induced differentiation. **a** The relative expression of Sox17 and BLIMP1 in POI-1-iPSCs and iPSCs-11 at day 4 and 8 in PGCs induction differentiation. **b** The percentages of CD38-positive cells and VASA-GFP-positive cells in culture system of POI-1-iPSCs and iPSCs-11 at days 4 and 8. **c** The relative expression of TFAP2C, NANOS3 and PRDM14 in induced system of POI-1-iPSCs and iPSCs-11 at days 4 and 8. **d** The percentage of VASA-positive cells at day 8 from POI-1-iPSCs, iPSCs-11, and iPSCs using DNMTi and not using DNMTi

Sox17 is the critical determinant for the inducted differentiation of human PGCs from competent human pluripotent stem cells, including human ESCs and iPSCs, and it initiates the human germ cell transcriptional network by work with upstream of BLIMP1 and other genes. The interaction of Sox17-BLIMP1 might be an important factor in human PGC specification and maintenance. The relative expression of Sox17 and BLIMP1 were analyzed during PGC induction differentiation in POI-1-iPSCs and iPSCs-11. The expression level of Sox17 and BLIMP1 both increased from day 4 to day 8. However, the expression level of Sox17 increased more obviously than BLIMP1 (Fig. 5a). CD38 is a key cell-surface marker of primordial germ cells. To determine if we obtained primordial germ cells from POI-1-iPSCs and iPSCs-11 during the late stage of induced differentiation, the CD38- and VASA-GFP-positive cells were sorted by fluorescence-activated cell sorting. The percentage of CD38-positive cells derived from POI-1-iPSCs were approximately 9.40 ± 1.45% and 35.52 ± 3.46% at day 4 and day 8; in the iPSCs-11 group, the percentage was 10.36 ± 1.38% and 38.21 ± 2.65%, respectively. The percentage of positive VASA-GFP cells derived from POI-1-iPSCs were approximately 18.45 ± 1.73% and 42.30 ± 2.65% at day 4 and day 8; in the iPSCs-11 group, the percentage was 16.82 ± 1.54% and 46.33 ± 3.18%, respectively (Fig. 5b). TFAP2C, NANOS3, and PRDM14 could be a marker for induction into the PGCLC from human iPSCs. The relative expression of three key genes was analyzed during PGC induction differentiation in POI-1-iPSCs and iPSCs-11 (Fig. 5c). These results showed that PGCs were induced from POI-1-iPSCs under our new induction system.

In our induced differentiation of PGCs from iPSCs, DNA methyltransferase inhibitor (DNMTi) was added at day 5 after pre-induction, which is different from the paper described [14]. In order to explore the efficiency of DNMTi on PGC induced differentiation from iPSCs, including iPSCs-11, POI-1-iPSCs, and iPSCs (normally established iPSCs from our lab) [13], we analyzed the percentage of VASA-positive cells at day 8 in our experiments using DNMTi and not using DNMTi. Our results showed that the percentage of VASA-positive cells in iPSCs-11, POI-1-iPSCs, and iPSCs using DNMTi was 46.33 ± 3.18%, 42.30 ± 2.65%, and 45.48 ± 2.62%, respectively. But in not using the DNMTi group, the percentage was 36.45 ± 2.19%, 35.57 ± 3.19%, and 38.58 ± 1.29%, respectively (Fig. 5d). These results indicated that DNA demethylation after pre-induction accelerated the PGC differentiation from human iPSCs.

### The induced PGCs from POI-1-iPSCs underwent epigenetic reprogramming

**Fig. 6:**
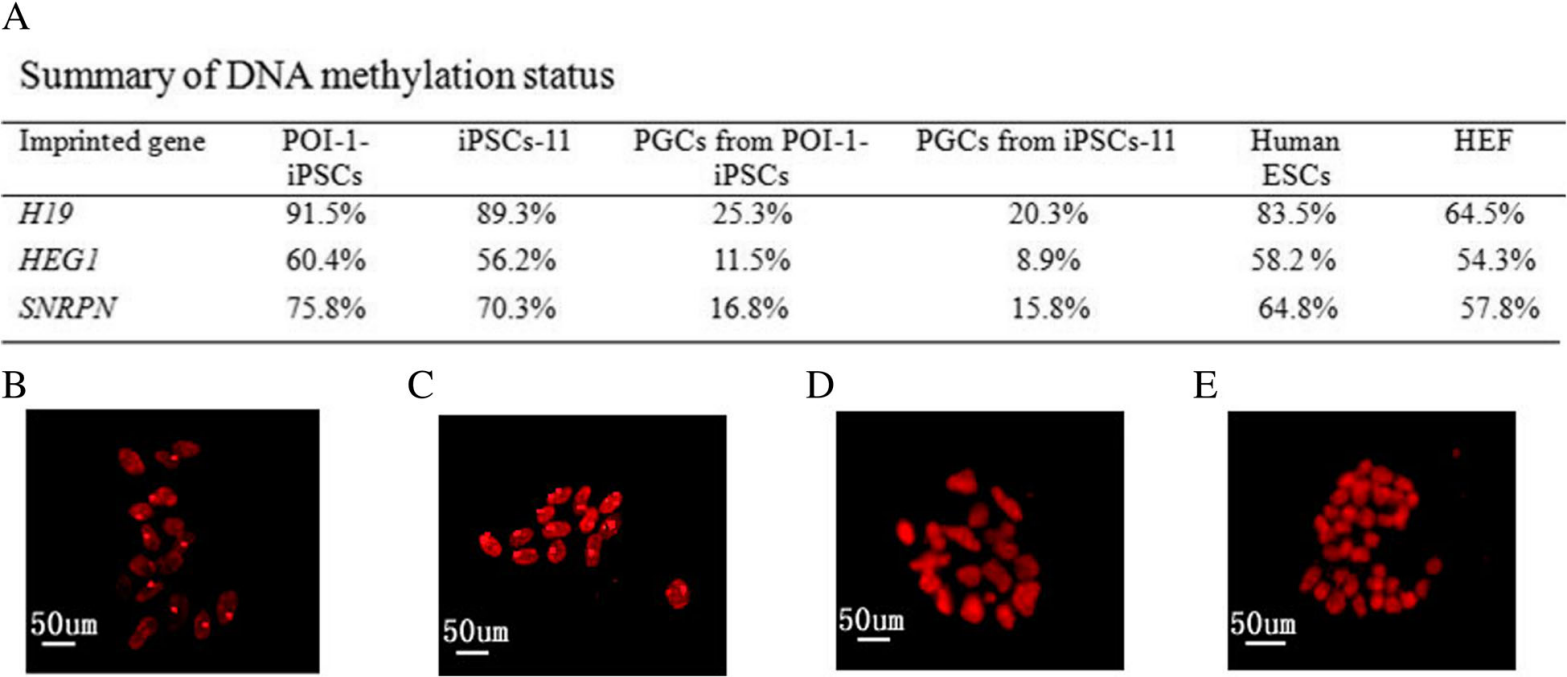
The dynamics of methylation and histone modifications in the induced PGCs from POI-1-iPSCs. **a** The CpGs of imprinted genes (*H19*, *PEG1*, and *SNRPN* DMRs) were analyzed following bisulfite sequencing from POI-1-iPSCs, iPSCs-11, PGCs from POI-1-iPSCs, PGCs from iPSCs-11, human ESCs, and HEF. Percentage methylation was calculated comparing the number of CpG methylated sites in undifferentiated iPSCs before differentiation with the number of CpG methylated sites at the same locus in the PGC. **b** Immunofluorescence for H3K9me3 (red) exhibit 100% (13/13) prominent H3k27me3 unclear body in differentiated human embryonic fibroblasts (EF), **c** 56.25% (9/16) prominent H3k27me3 unclear body in the induced PGCs from POI-1-iPSCs. While undifferentiated POI-1-iPSCs (**d**) and iPSCs-11 cells (**f**) showed diffuse staining

Recent studies have reported epigenetic reprogramming and genome-wide DNA demethylation dynamics in human germ cells [15–18]. Although the extent and the timing of DNA demethylation reported by these studies are somewhat different, the general consensus is that PGCs undergo epigenetic reprogramming that is similar overall to that seen in mouse PGCs. In our study, the induced PGCs from POI-1-iPSCs showed lower DNA methylation than POI-1-iPSCs, iPSCs-11, and human ESCs (Fig. 6a). In parallel, we also analyzed histone H3 lysine 27 tri-methylation (H3K27me3) by immunostaining and asked whether the Histone modifications had happened during PGC differentiation from pluripotent stem cells. Our results showed that the induced PGCs from POI-1-iPSCs displayed lower signals and lower levels of H3K9me3 (Fig. 6b) than human EF cells (Fig. 6c), but higher than undifferentiated POI-1-iPSCs (Fig. 6d) and iPSCs-11 (Fig. 6f), suggesting that the inactivated X chromosome was already reactivated in these induced PGCs. All the abovementioned results confirmed that these induced PGCs from POI-1-iPSCs had basic features of human PGCs in epigenetic status.

### The induced PGCs from POI-1-iPSCs had the potential for meiotic progression

**Fig. 7:**
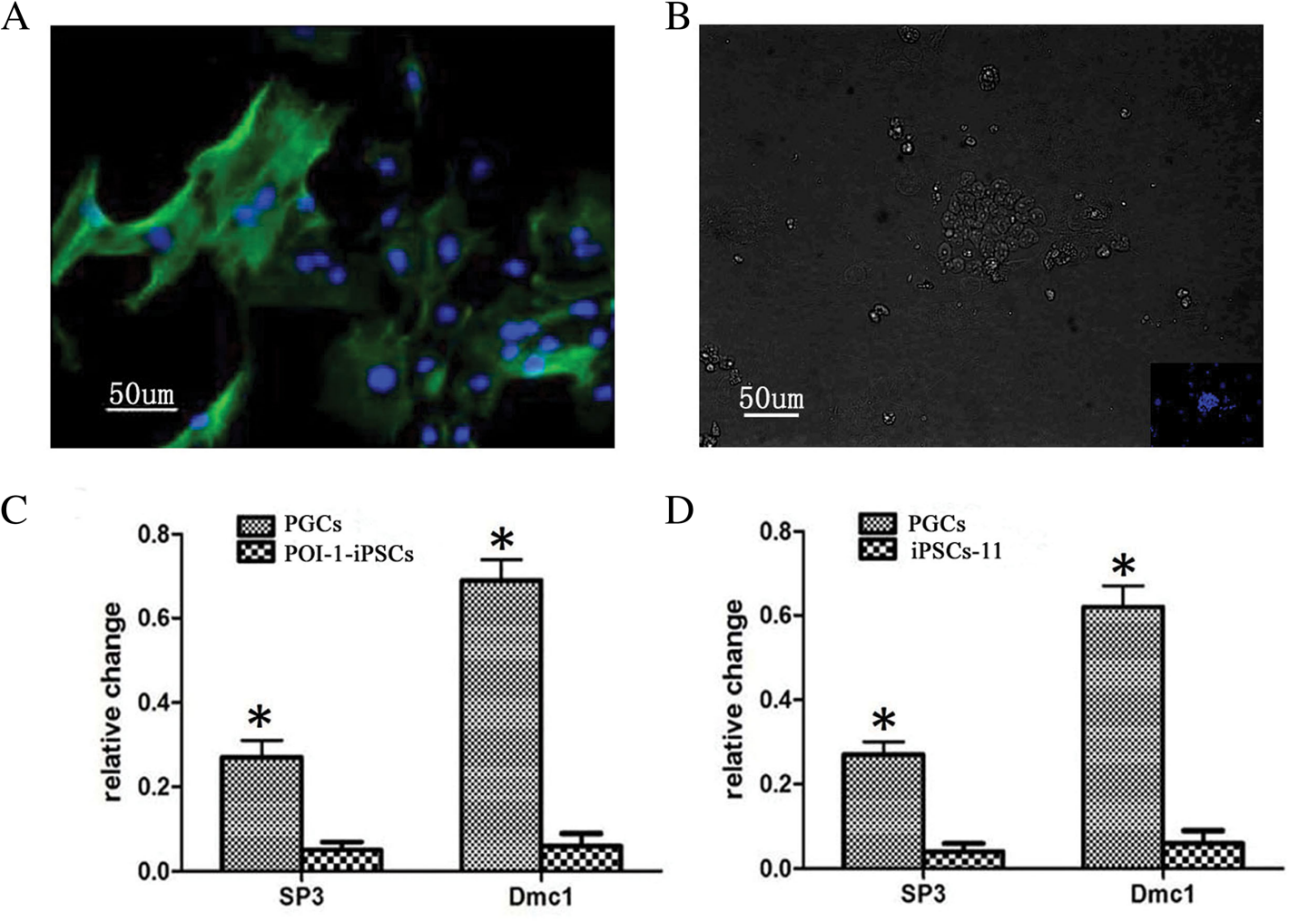
The induced PGCs from POI-1-iPSCs had the potential for meiotic progression. **a** The induced PGCs from POI-1-iPSCs at day 8 stained positively for SP3. **b** The POI-1-iPSCs stained negatively for SP3. **c** The relative expression level of SP3 and Dmc1 increased in induced PGCs from POI-1-iPSCs. **d** The relative expression level of SP3 and Dmc1 increased in induced PGCs from iPSCs-11

Above all, we verified that the PGCs induced from POI-1-iPSCs expressed special markers and epigenetic reprogramming status for human germ cells. We further performed a meiotic progression assay to confirm that induced human PGCs had the potential for meiotic progression in vitro. The meiotic recombination protein (Dmc1) and synaptonemal complex protein 3 (SP3) both are special markers for meiosis and play a central role in homologous recombination during meiosis [19]. We stained for SP3 positive in the induced PGCs from POI-1-iPSCs (Fig. 7a). But no signal was appeared in POI-1-iPSCs (Fig. 7b). The gene SP3 and Dmc1 were also expressed in the induced PGCs from POI-1-iPSC, not expressed in POI-1-iPSCs (Fig. 7c), and iPSCs-11 (no POI-iPSCs) (Fig. 7d). Our results showed that the PGCs exhibited such an ability, indicating that the induced PGCs derived from POI-1-iPSCs started to be meiotic at a late stage.

## Discussion

Herein, human POI-iPSCs with genotype of POI, including fragile X syndrome, abnormal karyotype (45, X; 45, X/46, XX; 45, XO and 47, XXX), and the gene mutation (*FIGLA* and *GDF9*), were generated using a lentiviral vector with four reprogramming factors under the 4i culture system. The established POI-1-iPSCs with the genotype of fragile X syndrome (CGG repeats) had similar characteristics to human ESCs, including a similar morphology, the expression profiles of pluripotency markers, the ability for long-term self-renewal, and the potential of differentiation into all three germ layers. Very importantly, the POI-1 iPSCs can be differentiated efficiently into human PGCs with the potential for meiotic progression.

Pluripotent states or germline competence is very important for the differentiation of PGCs from human iPSCs or ESCs. Most human iPSCs and ESCs are not real germline competent, which is the main reason that fewer studies have been published for the successful differentiation of human PGCs from iPSCs or ESCs. A recent study proved that changes in pluripotent cell states can be induced by environmental factors in respect to the gain and loss of competence for germ cell fate from human iPSCs and ESCs in the 4i culture system [15]. Some researcher had reported that human PGCs had underwent demethylation during the development period and the methylation dynamics influenced gene expression and germ cell fate [16, 17]. In our study, POI-iPSCs with germline competence were induced using the 4i culture system first, and pre-induction, and then DNA demethylation. Some researchers reported that human PGCs were induced from iPSCs in conditional medium and obtained VASA-positive cells [20]. However, the induction efficiency of human PGCs is relatively low (8.5 ± 0.29%). In our new culture system, positive VASA-GFP cells at day 8 after induction were 42.30 ± 2.65% in the population. The germline competence in the 4i culture system, suitable pre-induction, and then DNA demethylation are the main factors for human PGC induction with high efficiency. In conventional culture conditions, the competence of human iPSCs is rarely maintained and progressively lost.

There are two waves of global demethylation in human PGCs from the migrating stage to the gonadal stage in vivo. The global erasure of DNA methylation creates a super-hypomethylated germline genome [17]. It has also been confirmed that human iPSCs can differentiate into PGCs. But we did not know the methylation status of differentiated PGCs from iPSCs in vitro, and the relationship of DNA methylation and the induced differentiation of PGCs from iPSCs in vitro. In this study, we answered the two questions and confirmed that DNA demethylation accelerated the induction of PGCs from iPSCs in vitro. This provides a new and high-efficiency way for PGC induction differentiation from iPSCs in vitro.

POI is a complex disorder in reproductive-aged women. At the cell level, it is a disorder of oocyte development and function. The development and differentiation of PGCs, a precursor oocyte, also plays an important role in oocyte development and the physiopathology of POI patients. However, in the present study, the differentiation of PGCs and oocytes with a biological function from POI-iPSCs still needed to be further explored. Our study provided a new cell model and way to explore the pathophysiology of POI from cell development and differentiation in vitro at an early stage. Some studies have shown that the VASA-GFP reporter traces differentiated germ cells effectively in vitro. The stable transfection of the VASA-GFP reporter into ESCs or iPSCs has been reported based on the specificity of VASA as a germ cell marker in vivo [21]. In our study, we also established an effective system to trace the differentiation of germ cells in POI-iPSCs. At the same time, we believe that considerable work is still required in examining the differentiation from human PGCs to oocytes. Moreover, we have started exploring this area.

The lentiviral vectors could lead to insertional mutagenesis and increased tumorigenicity [22]. Furthermore, it could also reactivate some oncogenes, including *c-myc*. Some studies indicated that the activation of reprogramming factors could inhibit some cell differentiation of pluripotent stem cells, and at the same time, the reactivation of *oncogene*s can increase the risk of tumorigenicity [23]. In our study, lentiviral vectors were used for reprogramming iPSCs. We established POI-iPSCs with full potential and with the suitable integration of reprogramming factors. In our opinion, the quantity and the degree of the integration of reprogramming factors are very important. The proper integration of reprogramming factors is beneficial to pluripotency status. However, if there is more integration of the reprogramming factor, there is more potential for tumorigenicity. Some studies set up new cellular reprogramming strategies without using integrated vectors and support the new idea that patient cells could be reprogrammed without using integrating vectors, especially in future clinical applications. We recently reprogrammed a POI patient’s somatic cells using proteins and generated genomic modification-free, disease-specific iPS cells and are currently being analyzed to determine the differences between iPS cells made with lentiviral vectors and proteins.

iPS cell technology enables the generation of patient-specific pluripotent stem cells that carry patient/disease-associated genotypes. Disease-specific iPS cells have been produced from various diseases. These iPSCs provide unique opportunities to dynamically study disease-specific pathogenesis in vitro and in vivo. In this sense, the POI disease special iPSCs in our study, including fragile X syndrome (CGG repeats), abnormal karyotype (45, X; 45, X/46, XX; 45, XO and 47, XXX), and the gene mutation (*FIGLA* and *GDF9*) iPSCs would be useful for disease cell modeling, drug discovery, and eventually, autologous cell replacement therapies.

## Concussion

In summary, first, our study established some novel, systemic cell models for the studying of the pathogenesis of POI patients. Second, in our study, DNA demethylation may accelerate the induction of human PGCs from iPSCs in vitro, and the conclusion needs further exploration.

## Additional files

Additional file 1: **Table S1.** The information about the POI patients included in this study. (DOCX 18 kb)

Additional file 2: **Table S2.** Primers of PCR for bisulfite sequencing. (DOCX 15 kb)

Additional file 3: **Figure S1.** Genotype of iPSCs and PGCLCs. (DOCX 1039 kb)

## Abbreviations

AMH: Anti-Mullerian hormone
DAC: 5-Aza-2′-deoxycytidine
DNMTi: DNA methyltransferase inhibitor
EB: Embryonic body
EpiLCs: Epiblast-like cells
ESCs: Embryonic stem cells
H3K27me3: Histone H3 lysine 27 tri-methylation
iPSCs: Induced pluripotent stem cells
MEFs: Mouse embryonic fibroblasts
PGCs: Primordial germ cells
POI: Premature ovarian insufficiency
SP3: Synaptonemal complex protein 3

## References

[1] Christin-Maitre S, Braham R (2008). General mechanisms of premature ovarian failure and clinical check-up. Gynecol Obstet Fertil.

[2] Shelling AN (2010). Premature ovarian failure. Reproduction.

[3] Cox L, Liu JH (2014). Primary ovarian insufficiency: an update. Int J Women's Health.

[4] Fenton AJ (2015). Premature ovarian insufficiency: pathogenesis and management. J Midlife Health.

[5] Easley CA, Phillips BT, McGuire MM, Barringer JM, Valli H, Hermann BP, Simerly CR, Rajkovic A, Miki T, Orwig KE, Schatten GP (2012). Direct differentiation of human pluripotent stem cells into haploid spermatogenic cells. Cell Rep.

[6] Ramathal C, Durruthy-Durruthy J, Sukhwani M, Arakaki JE, Turek PJ, Orwig KE, Reijo Pera RA (2014). Fate of iPSCs derived from azoospermic and fertile men following xenotransplantation to murine seminiferous tubules. Cell Rep.

[7] Zhou Q, Wang M, Yuan Y, Wang X, Fu R, Wan H, Xie M, Liu M, Guo X, Zheng Y, Feng G, Shi Q, Zhao XY, Sha J, Zhou Q (2016). Complete meiosis from embryonic stem cell-derived germ cells in vitro. Cell Stem Cell.

[8] Hayashi K, Ogushi S, Kurimoto K, Shimamoto S, Ohta H, Saitou M (2012). Offspring from oocytes derived from in vitro primordial germ cell-like cells in mice. Science.

[9] Tesar PJ, Chenoweth JG, Brook FA, Davies TJ, Evans EP, Mack DL, Gardner RL, McKay RD (2007). New cell lines from mouse epiblast share defining features with human embryonic stem cells. Nature.

[10] Najm FJ, Chenoweth JG, Anderson PD, Nadeau JH, Redline RW, McKay RD, Tesar PJ (2011). Isolation of epiblast stem cells from preimplantation mouse embryos. Cell Stem Cell.

[11] Ying QL, Wray J, Nichols J, Batlle-Morera L, Doble B, Woodgett J, Cohen P, Smith A (2008). The ground state of embryonic stem cell self-renewal. Nature.

[12] Chan YS, Göke J, Ng JH, Lu X, Gonzales KA, Tan CP, Tng WQ, Hong ZZ, Lim YS, Ng HH (2013). Induction of a human pluripotent state with distinct regulatory circuitry that resembles preimplantation epiblast. Stem Cell.

[13] Yang S, Ding SF, Jiang XL, Sun BN, Xu QH (2016). Establishment and adipocyte differentiation of polycystic ovary syndrome-derived induced pluripotent stem cells. Cell Prolif.

[14] Irie N, Weinberger L, Tang WW, Kobayashi T, Viukov S, Manor YS, Dietmann S, Hanna JH, Surani MA (2015). Sox17 is a critical specifier of human primordial germ cell fate. Cell.

[15] Gafni O, Weinberger L, Mansour AA, Manor YS, Chomsky E, Ben-Yosef D, Kalma Y, Viukov S, Maza I, Zviran A, Rais Y, Shipony Z, Mukamel Z, Krupalnik V, Zerbib M, Geula S, Caspi I, Schneir D, Shwartz T, Gilad S, Amann-Zalcenstein D, Benjamin S, Amit I, Tanay A, Massarwa R, Novershtern N, Hanna JH (2013). The ontogeny of cKIT+ human primordial germ cells proves to be a resource for human germ line reprogramming, imprint erasure and in vitro differentiation. Nat Cell Biol.

[16] Gkountela S, Zhang KX, Shafiq TA, Liao WW, Hargan-Calvopiña J, Chen PY, Clark AT (2015). DNA demethylation dynamics in the human prenatal germline. Cell.

[17] Guo F, Yan L, Guo H, Li L, Hu B, Zhao Y, Yong J, Hu Y, Wang X, Wei Y, Wang W, Li R, Yan J, Zhi X, Zhang Y, Jin H, Zhang W, Hou Y, Zhu P, Li J, Zhang L, Liu S, Ren Y, Zhu X, Wen L, Gao YQ, Tang F, Qiao J (2015). The transcriptome and DNA methylome landscapes of human primordial germ cells. Cell.

[18] Tang WW, Dietmann S, Irie N, Leitch HG, Floros VI, Bradshaw CR, Hackett JA, Chinnery PF, Surani MA (2015). A unique gene regulatory network resets the human germline epigenome for development. Cell.

[19] Bishop DK, Park D, Xu L, Kleckner N (1992). DMC1: a meiosis-specific yeast homolog of E.coli recA required for recombination, synaptonemal complex formation, and cell cycle progression. Cell.

[20] Leng L, Tan Y, Gong F, Hu L, Ouyang Q, Zhao Y, Lu G, Lin G (2015). Differentiation of primordial germ cells from induced pluripotent stem cells of primary ovarian insufficiency. Hum Reprod.

[21] Kee K, Angeles VT, Flores M, Nguyen HN, Reijo Pera RA (2009). Human DAZL, DAZ and BOULE genes modulate primordial germ-cell and haploid gamete formation. Nature.

[22] Modlich U, Navarro S, Zychlinski D, Maetzig T, Knoess S, Brugman MH, Schambach A, Charrier S, Galy A, Thrasher AJ, Bueren J, Baum C (2009). Insertional transformation of hematopoietic cells by self-inactivating lentiviral and gammaretroviral vectors. Mol Ther.

[23] Deyle DR (2015). Generation of induced pluripotent stem cells. Methods Mol Biol.

