# Supplementary material for: Differentiation of primordial germ cells from premature ovarian insufficiency-derived induced pluripotent stem cells
Source: Stem Cell Res Ther. 2019 May 31;10:156. doi: 10.1186/s13287-019-1261-6 (PMC6545034; doi:10.1186/s13287-019-1261-6)
Supplement: Supplementary file 1 — Table S1. The information about the POI patients included in this study. (DOCX 18 kb) [file 13287_2019_1261_MOESM1_ESM.docx]

**Additional files**

Additional file 1: Table S1 The information about the POI patients included in this study

| Patient | Age | FSH  mIU/ml | AMH  (ng/ml) | Genetic characteristics | iPSCs |
| --- | --- | --- | --- | --- | --- |
| 1 | 26 | 38.8 | 0.06 | Fragile X syndrome: FMR1(CGG repeats) | POI-1-iPSCs, POI-2-iPSCs |
| 2 | 30 | 31. | 0.16 | Karyotype: 45,X | POI-3-iPSCs, POI-4-iPSCs |
| 3 | 26 | 70.0 | 0.32 | Karyotype: 45,X/46,XX | POI-5-iPSCs |
| 4 | 34 | 47.2 | 0.27 | The mutations of *FIGLA*( c.2 T > C) | POI-6-iPSCs |
| 5 | 39 | 23.3 | 0.41 | The mutations of GDF9( c.447C>T) | POI-7-iPSCs, POI-8-iPSCs |
| 6 | 22 | 80.1 | 0.23 | Karyotype : 47,XXX | POI-9-iPSCs |
| 7 | 25 | 56.1 | 0.28 | Karyotype: 45,XO | POI-10-iPSCs |
| No POI 1 | 25 | 7.1 | 3.05 | Karyotype: 46,XX | iPSCs-11 |
| No POI 2 | 29 | 5.3 | 4.84 | Karyotype: 46,XX | iPSCs-12 |
