# Supplementary material for: Differentiation of primordial germ cells from premature ovarian insufficiency-derived induced pluripotent stem cells
Source: Stem Cell Res Ther. 2019 May 31;10:156. doi: 10.1186/s13287-019-1261-6 (PMC6545034; doi:10.1186/s13287-019-1261-6)
Supplement: Supplementary file 2 — Table S2. Primers of PCR for bisulfite sequencing. (DOCX 15 kb) [file 13287_2019_1261_MOESM2_ESM.docx]

**Additional file 2**

Additional file 2 Table S2. Primers of PCR for bisulfite sequencing

| Gene | forward（5'-3'） | reverse（5'-3'） |
| --- | --- | --- |
| *H19* | tgt ata gta tat ggg tat ttt tgg agg ttt | tcc tat aaa tat cct att ccc aaa taa cc |
| *HEG1* | tyg ttg ttg gtt agt ttt gta ygg tt | aaa aat aac acc ccc tcc tca aat |
| *SNRPN* | ctc caa aac aaa aaa ctt taa aac cca aat tc | ggt ttt ttt tta ttg taa tag tgt tgt ggg g |
