# Supplementary material for: Differentiation of primordial germ cells from premature ovarian insufficiency-derived induced pluripotent stem cells
Source: Stem Cell Res Ther. 2019 May 31;10:156. doi: 10.1186/s13287-019-1261-6 (PMC6545034; doi:10.1186/s13287-019-1261-6)
Supplement: Supplementary file 3 — Figure S1. Genotype of iPSCs and PGCLCs. (DOCX 1039 kb) [file 13287_2019_1261_MOESM3_ESM.docx]

**Additional file 3**

Additional file 3 Figure S1. Genotype of iPSCs and PGCLCs

1. The targeted sequencing of iPSCs (POI-1-iPSCs and POI-2-iPSCs ) derived from patient 1.

Homo sapiens fragile X mental retardation 1 (FMR1), 88 CGG repeats on 5’ UTR

GCTCAGCTCCGTTTCGGTTTCACTTCCGGCGGCGGCGGCGGCGGCGGCGGCGGCGGCGGCGGCGGCGGCGGCGGCGGCGGCGGCGGCGGCGGCGGCGGCGGGGCGGCGGATGGAGGAGCTGGTGGTGGAAGTGCGGGGCTCCAAT
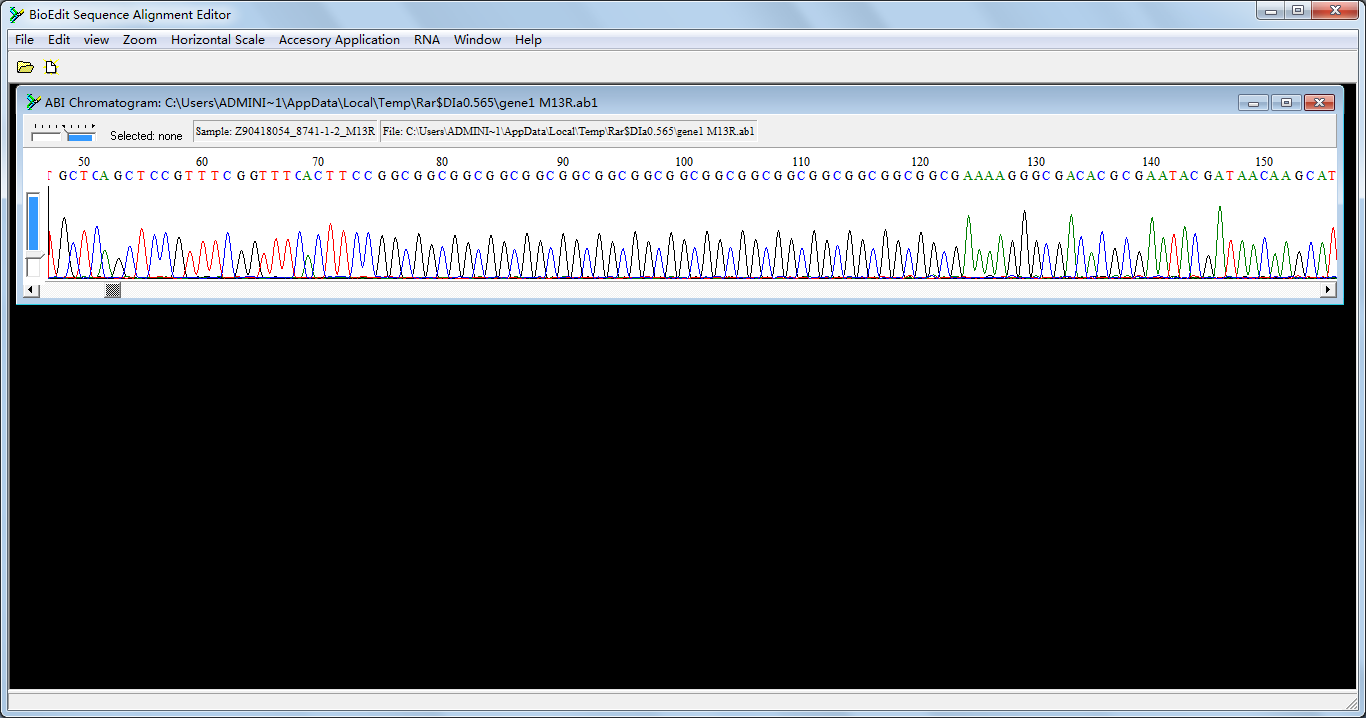


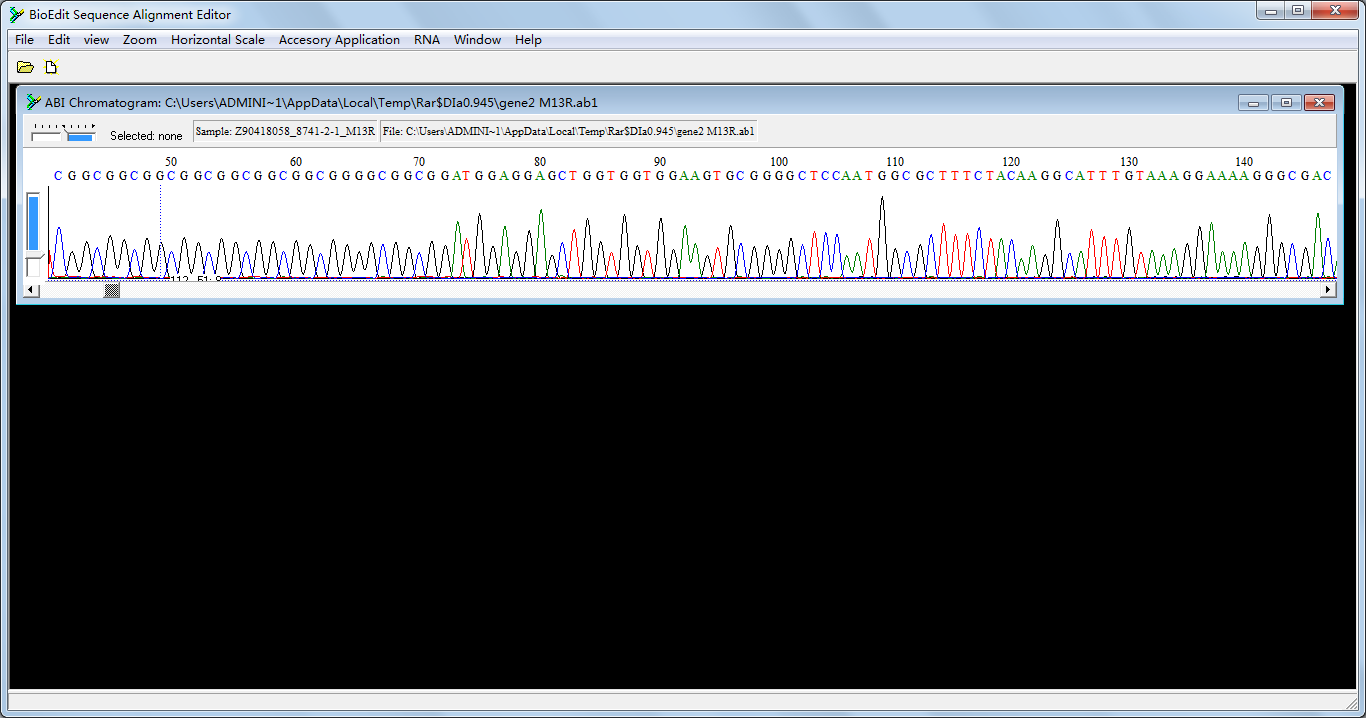


2. The targeted sequencing of induced PGCLCs from iPSCs (POI-1-iPSCs and POI-2-iPSCs ) derived from patient 1.

Homo sapiens fragile X mental retardation 1 (FMR1), 88 CGG repeats on 5’ UTR

GCTCAGCTCCGTTTCGGTTTCACTTCCGGCGGCGGCGGCGGCGGCGGCGGCGGCGGCGGCGGCGGCGGCGGCGGCGGCGGCGGCGGCGGCGGCGGCGGCGGGGCGGCGGATGGAGGAGCTGGTGGTGGAAGTGCGGGGCTCCAAT

**
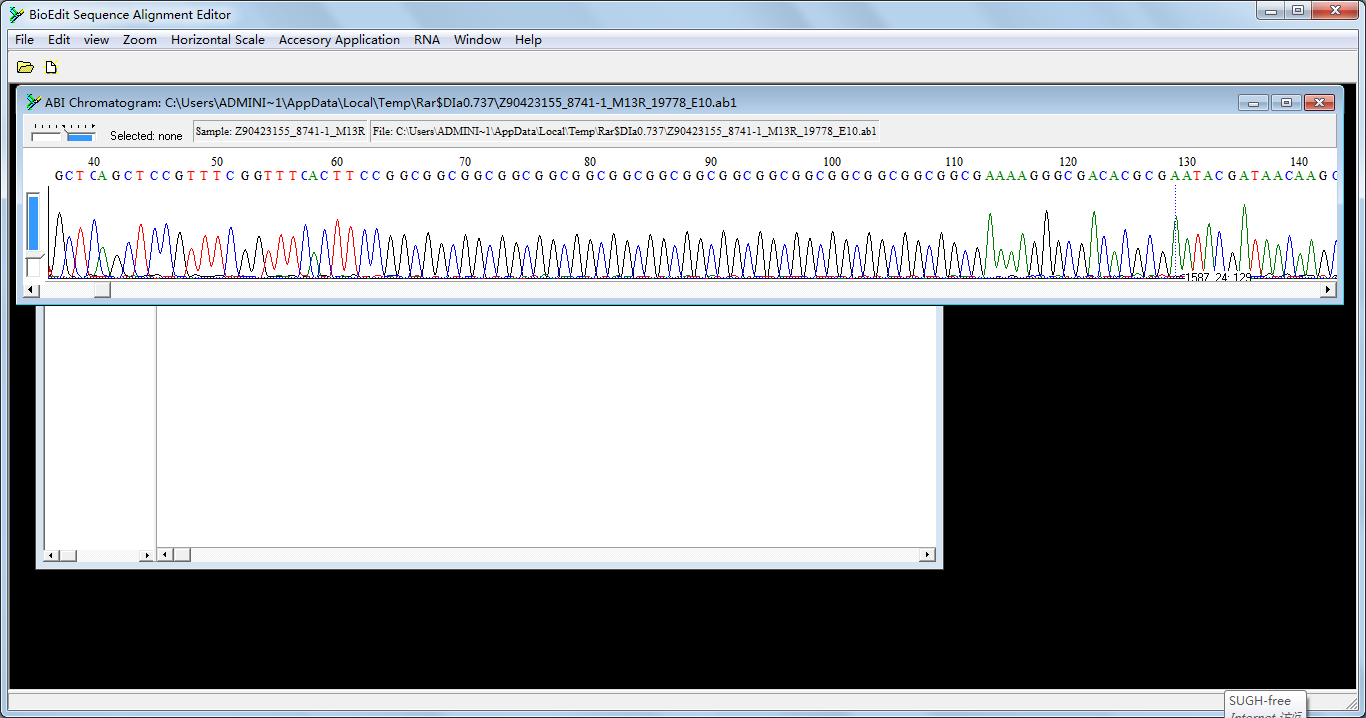
**

**
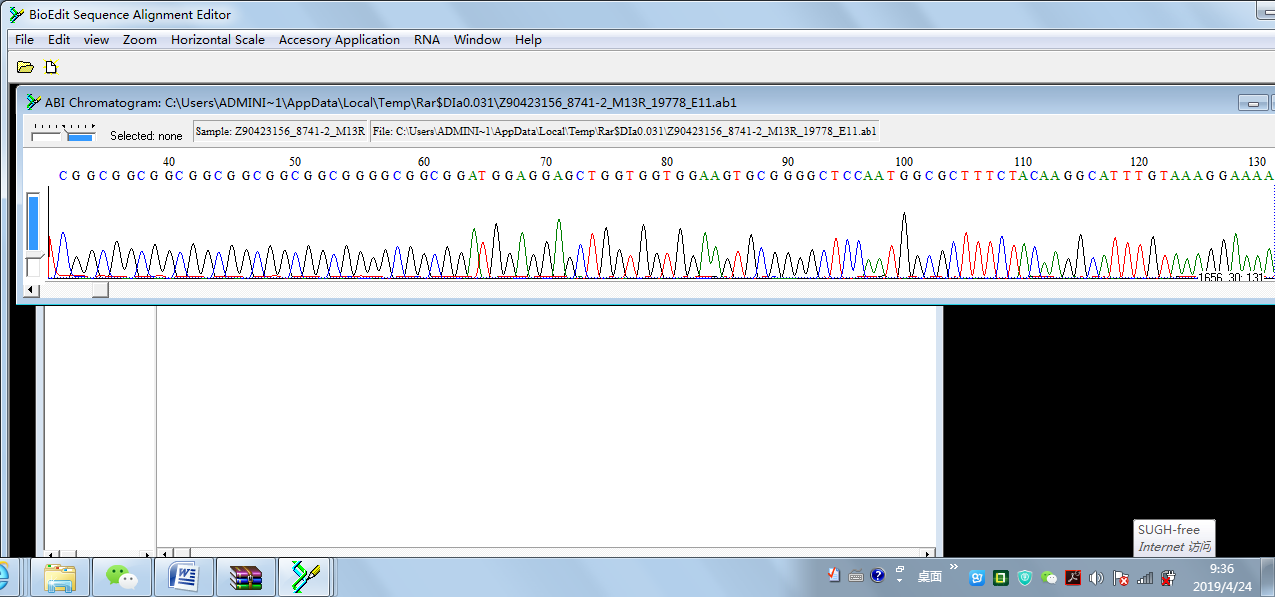
**

3. The karyotype of iPSCs (POI-3-iPSCs and POI-4-iPSCs) derived from patient 2.


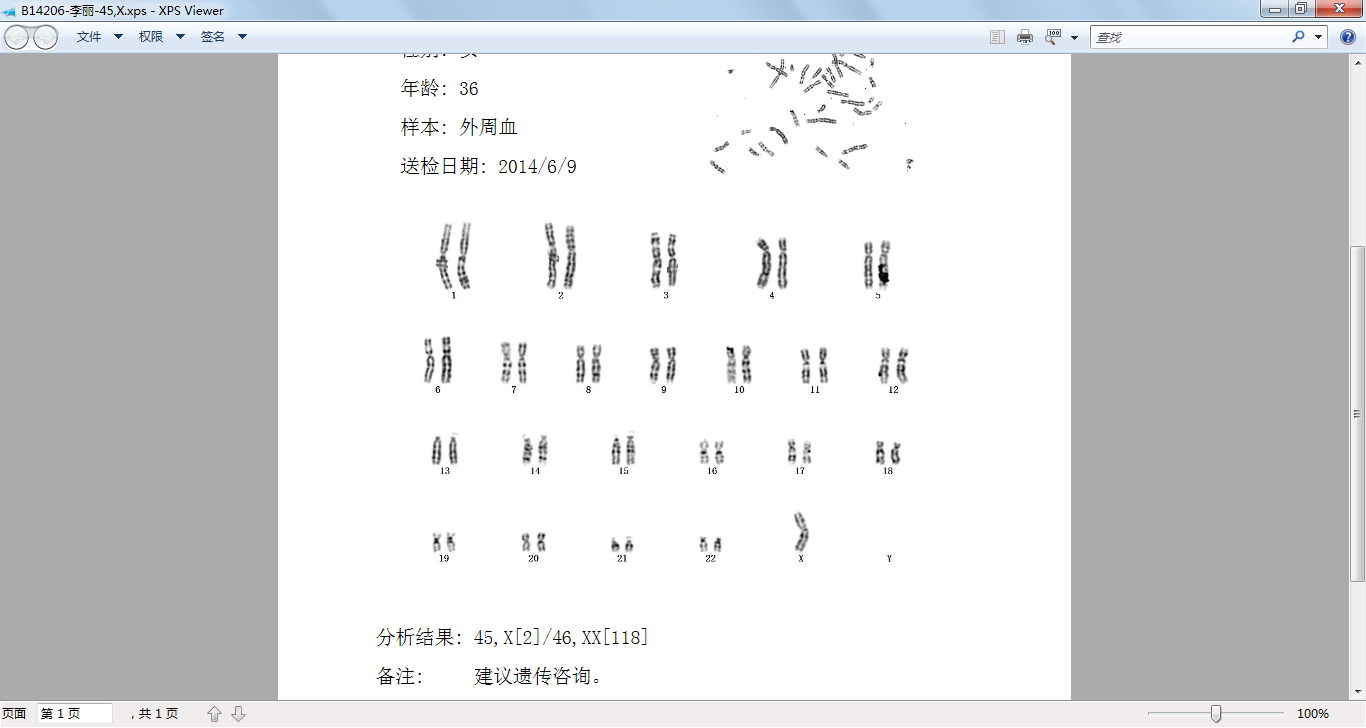


Karyotype: 45, X

4. The karyotype of induced PGCLCs from iPSCs (POI-3-iPSCs and POI-4-iPSCs) derived from patient 2.


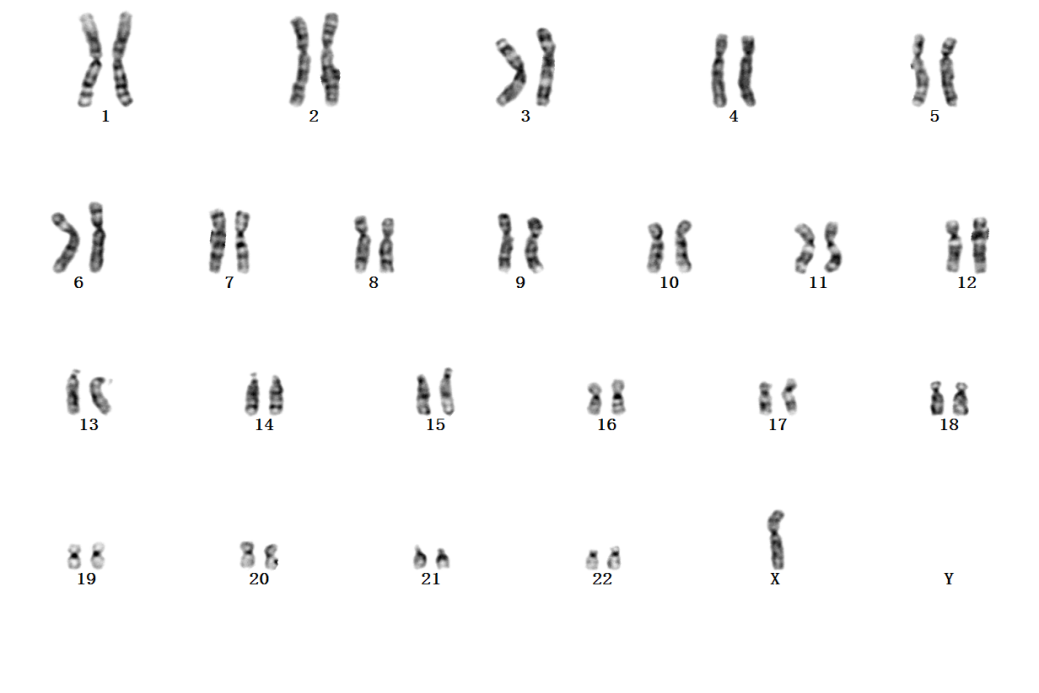


Karyotype: 45, X

5. The karyotype of iPSCs ( POI-5-iPSCs) derived from patient 3.


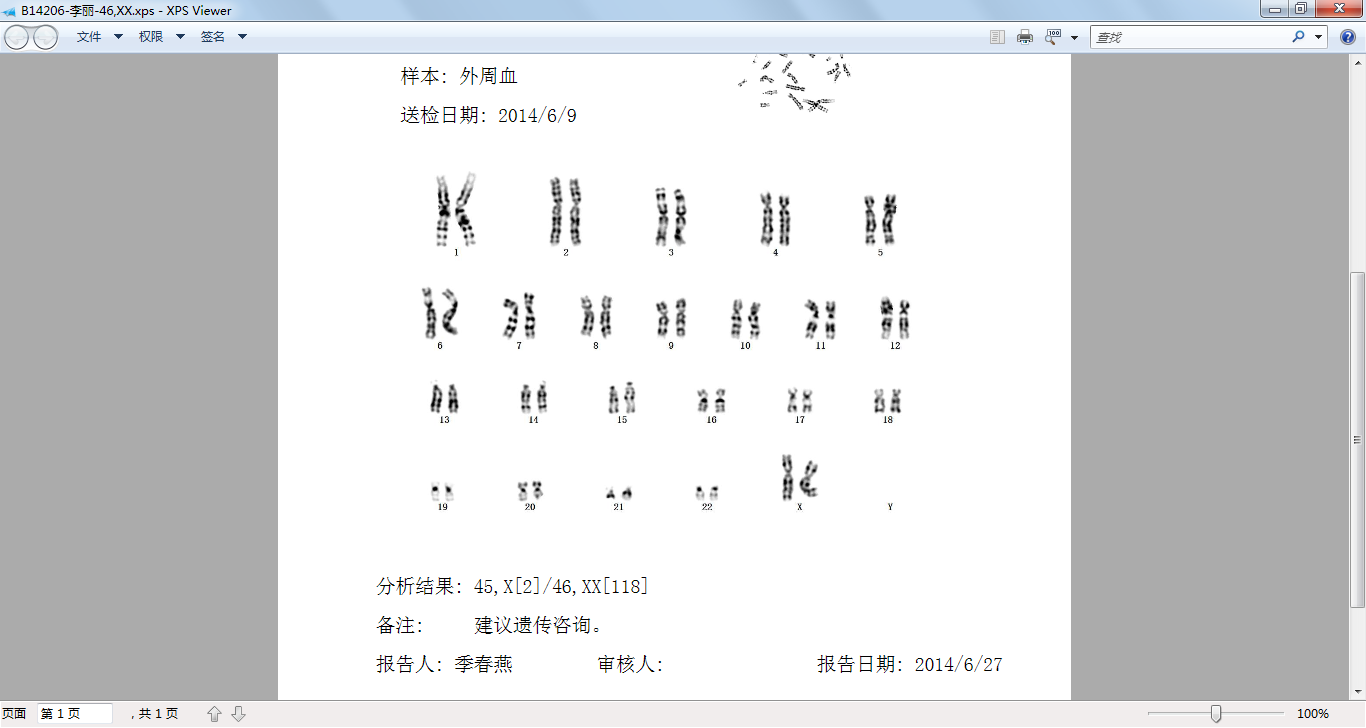


Karyotype: 46, XX

6. The karyotype of induced PGCLCs from iPSCs ( POI-5-iPSCs) derived from patient 3.


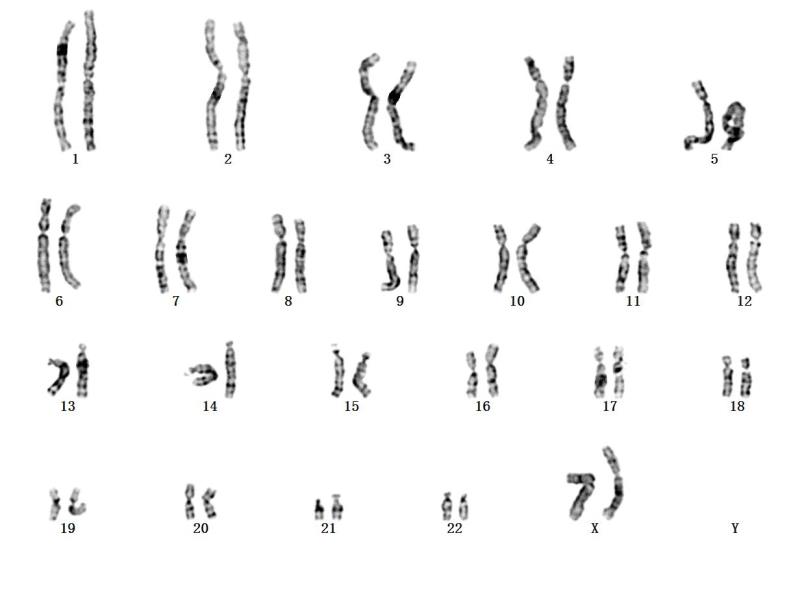


Karyotype: 46, XX

7. The targeted sequencing of iPSCs (POI-6-iPSCs ) derived from patient 4

*FIGLA* ( c.2 T >C)

GGCCTCAGCGGCCTCGGCCCTGCCACGGACCCCGCGCCCGGCGTCCTAGATC

5’UTR T CDS region

CCCGCGC


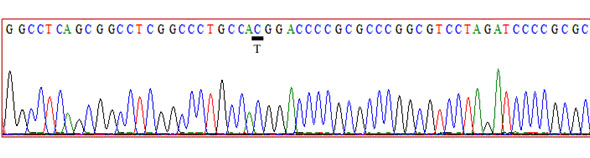


8. The targeted sequencing of induced PGCLCs from iPSCs (POI-6-iPSCs ) derived from patient 4

*FIGLA* ( c.2 T >C)

GGCCTCAGCGGCCTCGGCCCTGCCACGGACCCCGCGCCCGGCGTCCTAGATC

5’UTR T CDS region

CCCGCGC


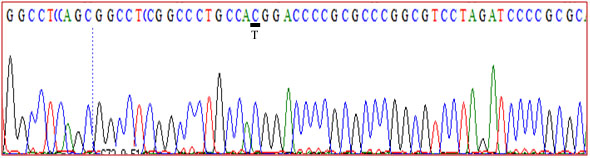


9. The targeted sequencing of iPSCs (POI-7-iPSCs and POI-7-iPSCs ) derived from patient 5.

GDF9 ( c.447C>T)

TCGCATTACTACTGTTGAACACTTACTCAAGTCAGTCTTGCTGTACAATATCA

ACAACTCA


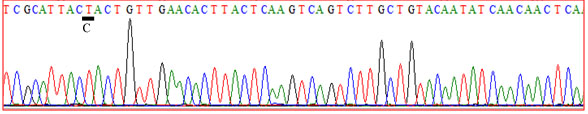


10. The targeted sequencing of induced PGCLCs from iPSCs (POI-7-iPSCs and POI-8-iPSCs ) derived from patient 5.

GDF9 ( c.447C>T)

TCGCATTACTACTGTTGAACACTTACTCAAGTCAGTCTTGCTGTACAATATCA

ACAACTCA


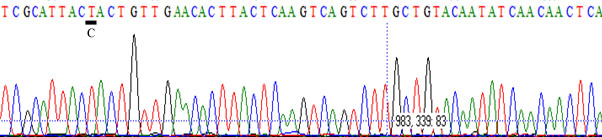


11. The karyotype of iPSCs (POI-9-iPSCs) derived from patient 6.


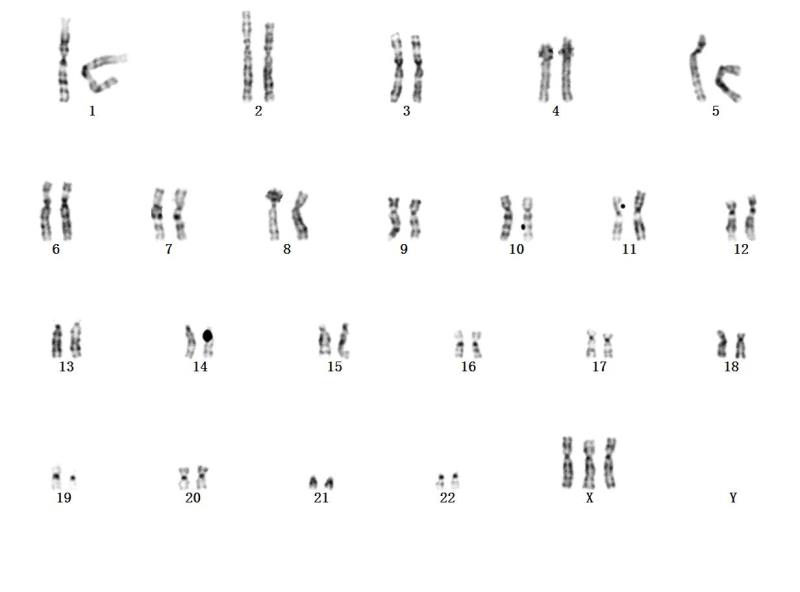


Karyotype: 46, XXX

12. The karyotype of induced PGCLCs from iPSCs (POI-9-iPSCs) derived from patient 6.


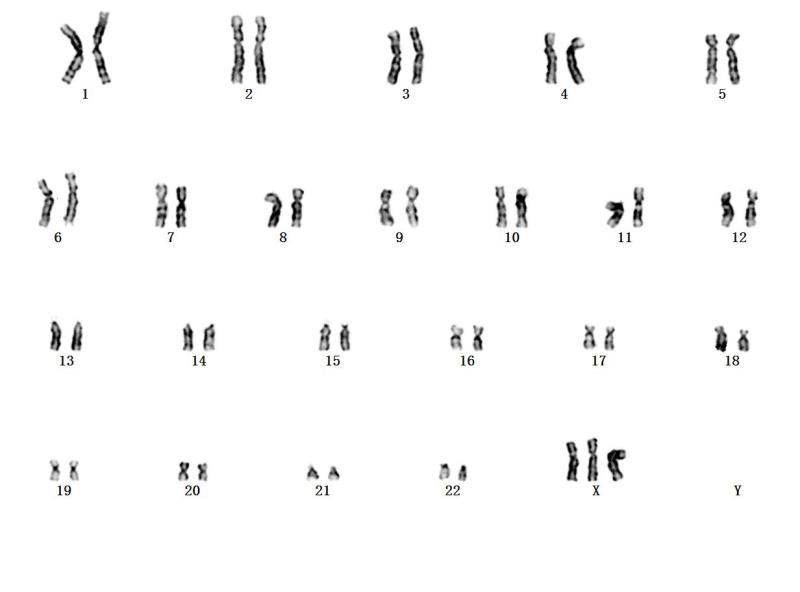


Karyotype: 46, XXX

13. The karyotype of iPSCs (POI-10-iPSCs) derived from patient 7


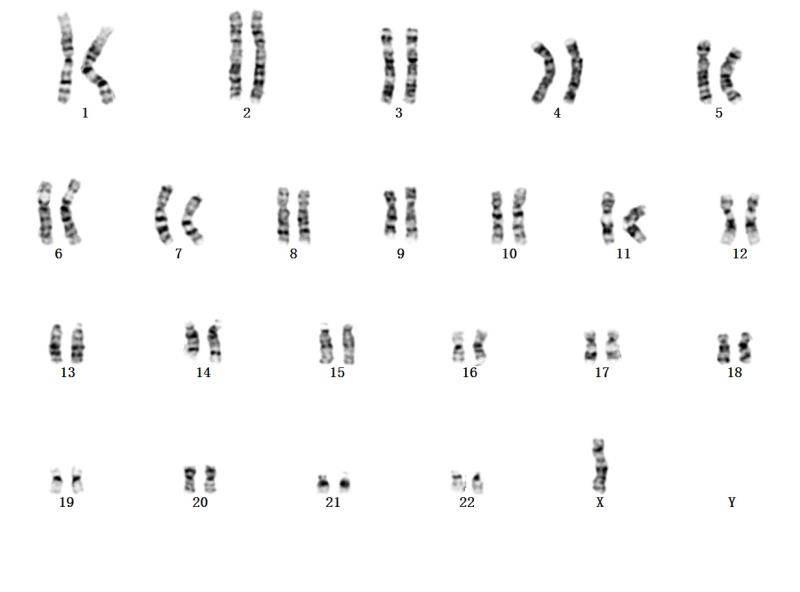


Karyotype: 45, XO

14. The karyotype of induced PGCLCs from iPSCs (POI-10-iPSCs) derived from patient 7


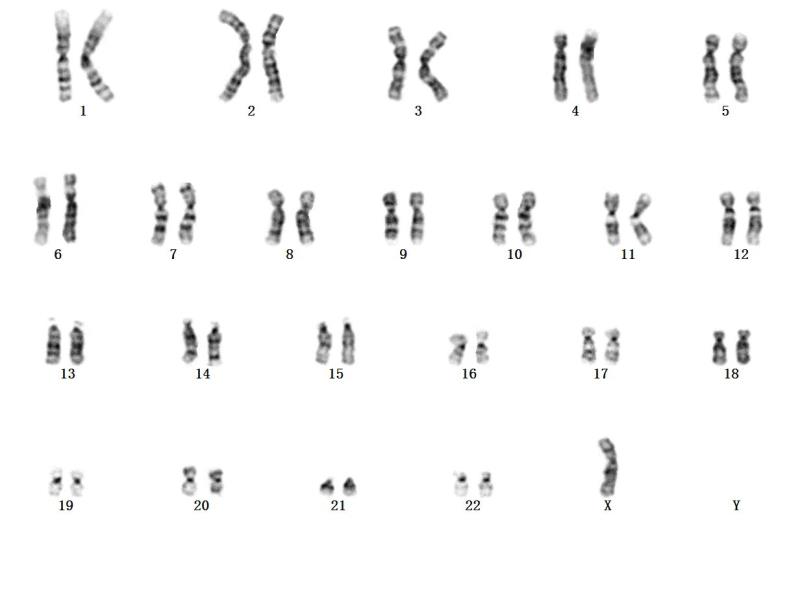


Karyotype: 45, XO
